# Supplementary material for: Social and Family Factors as Determinants of Sleep Habits in Japanese Elementary School Children: A Cross-Sectional Study from the Super Shokuiku School Project
Source: Children (Basel). 2021 Feb 5;8(2):110. doi: 10.3390/children8020110 (PMC7915430; doi:10.3390/children8020110)
Supplement: Supplementary file 1 [file children-08-00110-s001.pdf]

Supplementary file 1.

Research questionnaire in the Super Shokuiku School Project (Phase 3)

Name ________________ Sex ______

Grade _______________ Age ______

Child sleep habits (to child)

Q. What time do you go to bed?

Before 21:00, 21:00-21:30, 21:30-22:00, 22:00-22:30, 22:30-23:00, After 23:00

Q. What time do you wake up?

Before 6:00, 6:00-6:30, 6:30-7:00, 7:00-7:30, After 7:30

Q. How long do you usually sleep?

<7h, 7-8 hours, 8-9 hours, 9-10 hours, ≥10 hours

Q. Do you feel sleepy in the daytime?

No, Yes

Child lifestyles (to child)

Q. Do you have breakfast?

Every day, Usually, Sometimes, Skipping

Q. How long do you have screen time on a weekday, including television and film viewing, gaming, and Internet use?

None, <1, 1-2 hours, 2-3 hours, 3-4 hours, ≥4 hours

Q. How often do you exercise or play outside?

Very often, Often, Rarely, Almost never

Social background (to parents or guardians)

Q. Household

Grandfather, Grandmother, Father, Mother, Brothers and sisters, Other

Q. Family affluence

Very affluent, Affluent, Neither, Not much affluent, Not affluent

Q. Mother's employment status

Full-time, part-time, unemployed (housewife)

Parental lifestyle (to parents or guardians)

Q. Adequate sleep time

Yes, No

Q. Not smoking

Yes, No

Q. Appropriate weight control

Yes, No

Q. Not drinking excessively

Yes, No

Q. Regular physical activity

Yes, No

Q. Not skipping breakfast

Yes, No

Q. Not snacking frequently

Yes, No
